# Interplay between the alpharetroviral Gag protein and SR proteins SF2 and SC35 in the nucleus

**DOI:** 10.3389/fmicb.2015.00925

**Published:** 2015-09-08

**Authors:** Breanna L. Rice, Rebecca J. Kaddis, Matthew S. Stake, Timothy L. Lochmann, Leslie J. Parent

**Affiliations:** ^1^Division of Infectious Diseases and Epidemiology, Department of Medicine, Penn State College of MedicineHershey, PA, USA; ^2^Department of Microbiology and Immunology, Penn State College of MedicineHershey, PA, USA

**Keywords:** splicing factors, nuclear trafficking, SC35, SF2, retroviral Gag proteins, nuclear bodies

## Abstract

Retroviruses are positive-sense, single-stranded RNA viruses that reverse transcribe their RNA genomes into double-stranded DNA for integration into the host cell chromosome. The integrated provirus is used as a template for the transcription of viral RNA. The full-length viral RNA can be used for the translation of the Gag and Gag-Pol structural proteins or as the genomic RNA (gRNA) for encapsidation into new virions by the Gag protein. The mechanism by which Gag selectively incorporates unspliced gRNA into virus particles is poorly understood. Although Gag was previously thought to localize exclusively to the cytoplasm and plasma membrane where particles are released, we found that the Gag protein of Rous sarcoma virus, an alpharetrovirus, undergoes transient nuclear trafficking. When the nuclear export signal of RSV Gag is mutated (Gag.L219A), the protein accumulates in discrete subnuclear foci reminiscent of nuclear bodies such as splicing speckles, paraspeckles, and PML bodies. In this report, we observed that RSV Gag.L219A foci appeared to be tethered in the nucleus, partially co-localizing with the splicing speckle components SC35 and SF2. Overexpression of SC35 increased the number of Gag.L219A nucleoplasmic foci, suggesting that SC35 may facilitate the formation of Gag foci. We previously reported that RSV Gag nuclear trafficking is required for efficient gRNA packaging. Together with the data presented herein, our findings raise the intriguing hypothesis that RSV Gag may co-opt splicing factors to localize near transcription sites. Because splicing occurs co-transcriptionally, we speculate that this mechanism could allow Gag to associate with unspliced viral RNA shortly after its transcription initiation in the nucleus, before the viral RNA can be spliced or exported from the nucleus as an mRNA template.

## Introduction

Retroviruses are significant human and animal pathogens, causing cancer and immunodeficiency syndromes in a wide variety of species. Most well-known is the human immunodeficiency virus type 1 (HIV-1), the etiological agent of acquired immunodeficiency syndrome (AIDS). Many retroviruses that infect animals have served as important model systems for unraveling the mechanisms of retroviral replication, pathogenesis, and host defense. The first retrovirus discovered, the avian alpharetrovirus Rous sarcoma virus (RSV), has proven to be among the most valuable, launching challenges to existing dogmas that led to the discovery of reverse transcription and cellular oncogenes (reviewed in Parent, 2012).

Retroviruses are enveloped, positive-sense, single-stranded RNA viruses that package two copies of their genomes into virions. Following viral entry, the retroviral genomic RNA (gRNA) undergoes reverse transcription to generate a complementary, double-stranded DNA that integrates into the host cell genome to form the provirus. The integrated provirus is used as a template for the cellular RNA polymerase II to direct the synthesis of retroviral RNA. The genome-length retroviral transcript may be spliced to create subgenomic mRNAs, which are exported from the nucleus to synthesize other viral proteins. Alternatively, the full-length RNA may remain unspliced with two potential outcomes: it may serve as the mRNA template for the translation of the Gag and Gag-Pol structural proteins or it may be bound by the Gag protein for packaging into new virions as the gRNA. The Gag protein selects the gRNA for encapsidation through a high-affinity interaction between the nucleocapsid (NC) domain of Gag and the psi (Ψ) packaging sequence in the 5′ untranslated region of the viral RNA (Shank and Linial, 1980; Aronoff and Linial, 1991; Aronoff et al., 1993; Berkowitz et al., 1995, 1996; Butsch and Boris-Lawrie, 2002; Lee et al., 2003; Lee and Linial, 2004; Zhou et al., 2005, 2007).

Historically, it was thought that the initial Gag-gRNA interaction occurred in the cytoplasm or at the plasma membrane, where budding virions are released. Mounting evidence, including recent studies using sensitive microscopic imaging techniques, indicates that the Gag proteins of several retroviruses including HIV-1, RSV, mouse mammary tumor virus (MMTV), feline immunodeficiency virus (FIV), prototype foamy virus (PFV), Mason-Pfizer monkey virus (MPMV), and murine leukemia virus (MLV) undergo nuclear localization (Nash et al., 1993; Schliephake and Rethwilm, 1994; Amendt et al., 1995; Risco et al., 1995; Scheifele et al., 2002; Tobaly-Tapiero et al., 2008; Prizan-Ravid et al., 2010; Müllers et al., 2011; Renault et al., 2011; Elis et al., 2012; Kemler et al., 2012; Beyer et al., 2013; Lochmann et al., 2013). In the case of RSV, a connection has been established between Gag nuclear trafficking and gRNA incorporation. Genetic experiments demonstrated that targeting an RSV Gag mutant strongly to the plasma membrane reduced its nuclear trafficking, leading to the production of virus particles that encapsidate significantly reduced levels of gRNA (Scheifele et al., 2002). However, inserting an exogenous nuclear localization signal (NLS) into this Gag mutant restores gRNA packaging to nearly normal levels (Garbitt-Hirst et al., 2009). These results raise the intriguing possibility that nucleocytoplasmic transport of RSV Gag is required for proficient packaging of gRNA.

**Figure 1:**
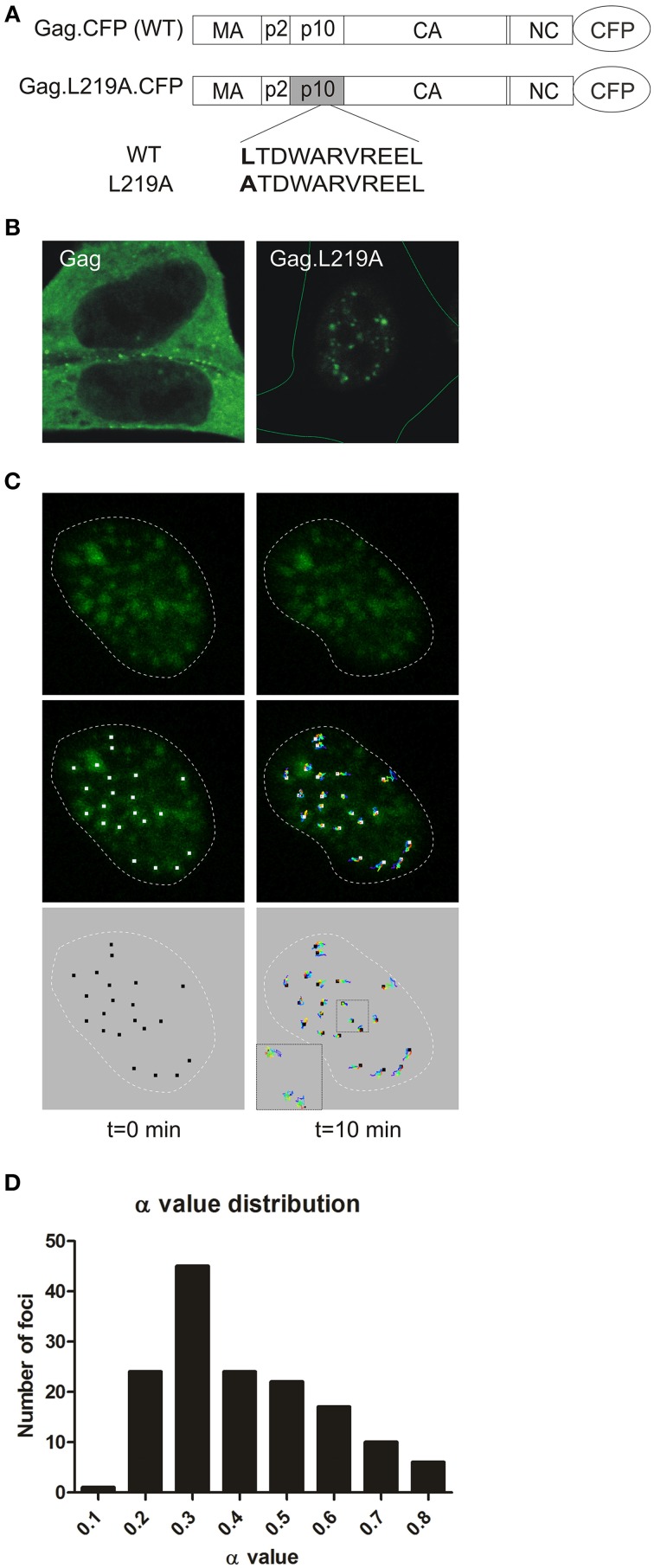
**Characterization of Gag.L219A nuclear foci. (A)** Schematics of the CFP-tagged wild-type RSV Gag protein (top) and the Gag.L219A nuclear export mutant (bottom), with leucine 219 in the p10 domain mutated to alanine. **(B)** Confocal micrographs of fluorescent protein-tagged wild-type Gag (left panel) and Gag.L219A (right panel) in QT6 cells. On the right hand image, the entire cell is outlined with a thin green line. **(C)** QT6 cells expressing Gag.L219A-CFP were imaged using time-lapse 3D confocal microscopy. A series of single optical slices through the nucleus were captured every 8 s for 10 min. After acquisition, the images were reconstructed as a 3D time-course using Imaris imaging software, and a single representative nucleus is shown with time = 0 on the left and *t* = 10 min on the right. The nucleus of each cell is outlined by a white dashed line. In the top panels, Gag foci (green) are shown. In the middle panels, the particle tracks were superimposed on the Gag foci, with white squares placed at the center of each focus (left) and tracks colored from blue (time = 0) to red (*t* = 10 min) in the middle panels. The particle tracks alone are shown in the bottom panel. In the lower left corner of the right image, a higher magnification of the particle tracks shows the course of the particles over the entire time period. **(D)** A histogram representing the anomalous diffusion coefficient α values for 149 nuclear foci is provided.

Treatment of RSV Gag expressing cells with the CRM1 inhibitor leptomycin B (LMB) traps Gag in the nucleus, and genetic mapping studies revealed a nuclear export signal (NES) in the p10 domain (Figure 1A). Mutation of hydrophobic residues within the NES causes Gag to accumulate in numerous, discrete nucleoplasmic foci and within nucleoli (Scheifele et al., 2002, 2005; Kenney et al., 2008; Lochmann et al., 2013). These nucleoplasmic foci are also observed at a lower frequency in the nuclei of cells expressing the wild-type Gag protein in the absence of LMB treatment (Figure 1B), providing evidence that formation of nuclear foci cannot be completely attributed to drug treatment or mutation. Furthermore, we demonstrated that Gag NES mutant proteins remain assembly-competent, as they interact with wild-type Gag proteins and can be rescued into virus particles (Kenney et al., 2008). The number and size of Gag nuclear foci increase with higher protein expression levels of the NES mutant Gag protein (data not shown), therefore it is possible that smaller accumulations of wild-type Gag proteins may form at lower expression levels, but these small foci are not readily detected by imaging studies.

To characterize the intranuclear population of RSV Gag proteins, we undertook the present studies to determine whether Gag nuclear foci share properties with host proteins that accumulate in nuclear bodies. These well-characterized subnuclear bodies are dynamic, non-membrane bound structures where nuclear proteins that perform specific functions are concentrated (Dundr and Misteli, 2010; Mao et al., 2011), including nuclear speckles, paraspeckles, and promyelocytic leukemia (PML) bodies. Nuclear speckles store and modify splicing factors that process pre-mRNAs (Mintz and Spector, 2000; Spector and Lamond, 2011). Paraspeckles are nucleated by the binding of the PSP1 protein to the long noncoding RNA NEAT1 and function in the retention of incompletely or aberrantly processed mRNAs (Fox et al., 2002; Prasanth et al., 2005; Bond and Fox, 2009; Clemson et al., 2009; Fox and Lamond, 2010; Souquere et al., 2010; Naganuma and Hirose, 2013; Yamazaki and Hirose, 2015). PML bodies form in response to DNA damage, stress, and viral infection (Dundr and Misteli, 2010; Mao et al., 2011). In this report, we examined whether the nuclear foci formed by nuclear-restricted Gag proteins have features in common with nuclear bodies and whether Gag localizes to any of the same nucleoplasmic sites as components of host nuclear bodies.

## Materials and methods

### Expression vectors

RSV Gag expression plasmids: pGag-GFP (Scheifele et al., 2002), pGag.L219A-CFP, pGag.L219A-YFP were described previously (Kenney et al., 2008). Expression plasmids used to encode human nuclear body proteins PSF and p54/nrb were constructed using PCR to exchange YFP for GFP from plasmids pGFP-PSF and pGFP-p54nrb, which were gifts from Dr. James Patton (Dye and Patton, 2001; Peng et al., 2002); human pSC35-YFP and human pYFP-SF2/ASF were gifts from Dr. David Spector (Prasanth et al., 2003); human pYFP-SUMO1 and human pCFP-PML were gifts from Dr. Mary Dasso (Ayaydin and Dasso, 2004); human pYFP-PSP1 was a gift from Dr. Angus Lamond, University of Dundee, UK; and murine pGFP-Clk1 was a gift from Alan Cochrane (Wong et al., 2011) (with permission from John Bell, University of Ottawa), in which GFP was exchanged with mCherry using PCR amplification and restriction fragment exchange.

### Cells, transfections, fixation, and immunofluorescence

QT6 cells, chemically transformed quail fibroblasts (Moscovici et al., 1977), were maintained as described (Craven et al., 1995), seeded at 0.2 × 10^6^ on coverslips in 35 mm dishes containing glass coverslips and transfected using the calcium phosphate method (Fujiwara et al., 1988) with the following plasmids: pGag.L219A-CFP (1.5 μg), pGag-CFP (500 ng), pCMV.SC35-YFP (100 and 125 ng), pCMV.YFP-SF2 (125 ng), pYFP-PSP1 (100 and 125 ng), pYFP-Nrb (100 ng), and pYFP-PSF (100 ng). Cells were fixed 16 h post-transfection in 3.7% PFA in PHEM buffer (120 mM PIPES, 55 mM HEPES, 20 mM EGTA, and 16.5 mM MgSO_4_, pH to 7.0) (Matic et al., 2008) for 10 min, incubated with DAPI at 5 μg/ml for 1 min and mounted on slides in SlowFade Antifade mounting medium (Invitrogen).

HeLa cells (Azad et al., 1993) were maintained as described (Lochmann et al., 2013), seeded at 0.4–0.5 × 10^6^ on coverslips in 35 mm dishes containing glass coverslips, and transfected using Lipofectamine 2000 (Invitrogen) with the following plasmids: pGag.L219A-CFP (4 μg), pCMV.SC35-YFP (1 μg), pCMV.YFP-SF2 (500 ng), pYFP-PSP1 (250 ng), pYFP-nrb (250 ng), and pYFP-PSF (250 ng). Cells were fixed between 18 and 23 h post-transfection with 3.7% PFA in PHEM for 10 min at room temperature, stained with DAPI at 5 μg/ml for 1 min, and mounted on slides in SlowFade Antifade mounting medium (Invitrogen). To detect endogenous phosphorylated RS domain proteins, cells were fixed in 3.7% PFA in PHEM for 10 min, permeabilized with 0.25% Triton X-100 for 10 min at room temperature, blocked with 10% BSA in PBS for 1 h, incubated for 1 h with mouse anti-SC35 (Sigma S4045) antibody at a dilution of 1:1000, which recognizes the phosphorylated RS domains of the splicing factors SC35 and SF2, in 3% BSA/0.01% Tween-20 in PBS, and incubated with donkey anti-mouse Alexa 647 (Invitrogen) at a 1:1000 dilution for 1 h at room temperature.

### Laser-scanning confocal microscopic imaging

Cells were imaged using a Leica SP8 TCS scanning confocal microscope equipped with a White Light Laser (WLL) and argon laser using a 63X oil immersion objective. Sequential scanning between frames was used to average four frames for each image. DAPI was excited with the 405 nm UV laser at 10% laser power and emission detection between 415 and 450 nm using a PMT detector. CFP was imaged using the WLL excited with the 470 nm laser line and a hybrid detector window of 475–500 nm. YFP was imaged using the WLL with a laser line excitation of 514 nm and a hybrid detector window of 518–650 nm. Alexa 647 was imaged using the WLL with the 647 laser line and the hybrid detector window ranged from 652 to 775 nm. mCherry was imaged using the 587 nm laser line and the hybrid detector window of 592–637 nm. All channels using the hybrid detectors had a time gating of 0.3 to 6.5 ns.

### Particle tracking in living cells

QT6 cells seeded at a density of 0.2 × 10^6^ were cultured in 35-mm glass-bottomed dishes (MatTekCorporation) and transfected with 1.5 μg pGag.L219A-CFP. Cells were imaged on a live cell stage equilibrated to 38.5°C with 5% CO_2_ at 16 h post-transfection using a Leica SP8 TCS scanning confocal microscope to capture 3D time-lapse images with a 63X water objective. Imaging was performed with a line average of two using the WLL with a laser line excitation of 470 nm and a hybrid detector window of 475–601 nm. A series of 0.3 μm ocular slices were captured to create a z-stack encompassing the entire nucleus approximately every 8 s for 10 min at 1400 Hz. The captured data was imported into Imaris analysis software (v8.0 Bitplane) to create a 3D volume rendering. Using the Imaris spot tool, Gag.L219A-CFP foci that measured ≤650 nm in diameter were identified in 3D space and tracked using Brownian motion detection. Correction for 3D drift was applied within the software, and only foci that were identified during the entire 10 min time-lapse period were analyzed. After focal drift compensation was performed, a line representing the movement of each individual particle was then superimposed onto the 3D time-lapse images. The Imaris software reported statistics for individual and average particle movement, and the x, y, z positions for each individual particle at each time point was exported from Imaris into MatLab using a script publically available at https://bitbucket.org/tim_lochmann/imaris-parser-for-msdanalyzer. The mean squared displacements (MSD) over time and diffusion coefficients were analyzed using the MSD analyzer script package (Tarantino et al., 2014). The MSD over time for each focus was calculated, and curves were fitted to the data. The α (anomalous diffusion coefficient) was calculated for 149 foci that had a curve fit of *R*^2^ > 0.8. The binned α values were displayed as a histogram. The value for α was used to determine the type of mobility of each particle as defined by these parameters: α < 0.1, confined diffusion; 0.1 ≤ α < 0.9, obstructed diffusion; 0.9 ≤ α < 1.1, simple diffusion; and α ≥ 1.1, directed motion (Bacher et al., 2004).

### Quantitative image analyses

To quantitatively analyze co-localization of fluorescent proteins in cells, ImageJ (v1.49p, Schindelin et al., 2012) was used to calculate Mander's statistics using the Just Another Colocalization Plugin (JACoP) (Bolte and Cordelières, 2006). A minimum of 8 QT6 cells and 4 HeLa cells were analyzed per condition, the mean ± standard error of the mean for each Mander's score was calculated, and the values were analyzed statistically using a two-tailed, unpaired *t*-test. Outliers were determined and removed using the Grubbs test using α = 0.05. (http://graphpad.com/quickcalcs/Grubbs1.cfm). Anti-Phospho RS domain staining was quantitatively analyzed using ImageJ by measuring the mean signal intensity of the nucleus for the antibody channel in cells with or without Gag.L219A and statistical analysis was performed as described above.

For quantification of nuclear foci, QT6 cells were seeded on coverslips at 0.4 × 10^6^ and transfected with 1.5 μg of pGag.L219A-CFP and 125 ng of plasmids expressing YFP-tagged SF2, SC35, or PSP1 using the calcium phosphate method. DF1 cells (Himly et al., 1998) were seeded on coverslips at a density of 0.6 × 10^6^, and co-transfected with 4 μg of pGag.L219A-CFP and 1 μg of each plasmid expressing a YFP-tagged host nuclear factor (SC35, SF2, or PSP1) using FuGene HD (Promega). Sixteen hours post-transfection, cells were fixed with 3.7% paraformaldehyde in PHEM buffer for 10 min at room temperature and mounted on slides with SlowFade Antifade mounting medium. Images were captured with a Deltavision DV Elite microscope (Applied Precision) using a 60 × (DF1 cells) or 100 × (QT6 cells) oil immersion objective with *N* = 1.514 oil using a CoolSNAPHQ2 (Photometrics) camera. Images were deconvolved and composite channel images were exported as RGB TIFF files post-acquisition using softWorx (v5.5.1, Applied Precision). ImageJ (v1.49o) (Schneider et al., 2012) was used for downstream export and analysis. A macro (modified from Alex Herbert, “ImageJ Batch Processing.” http://www.sussex.ac.uk/gdsc/intranet/pdfs/ImageJBatchProcessing.pdf) was used to split, recolor, and save the composite channel image into the constituent CFP and YFP channel images. The section through the widest diameter of the nucleus was cropped from a single optical slice obtained from a minimum of 20 DF-1 cells and 16 QT6 cells. The number of Gag.L219A-CFP nuclear foci were counted using the same modified macro, which first automatically adjusted the histogram of each image from 1 to the maximum pixel intensity value of that image, and then applied a pixel intensity threshold determined empirically for each slide. Foci were identified with a size constraint of ≥4 pixels squared and with no circularity constraint. Outliers were removed, as determined by the Grubbs test using α = 0.05. Prism statistical package (GraphPad Software 5.04) was used to create scatter plots showing the mean and standard error of the mean for the number of Gag nuclear foci, and a two-tailed, unpaired *t*-test was performed.

## Results

### RSV Gag.L219A nuclear foci exhibit obstructed diffusion

The fluorescently-tagged wild-type RSV Gag protein (Gag-GFP) localizes primarily to the plasma membrane with the visualization of several small foci in the nucleus when examined using confocal microscopy (Figures 1A,B, top left panel). By contrast, the Gag L219A mutant (Gag.L219A-CFP), which contains a single amino acid change that inactivates the NES in the p10 domain, is predominantly localized to the nucleus within numerous, discrete, punctate foci (Figures 1A,B, top right panel) (Scheifele et al., 2005). As previously described, Gag.L219A also concentrates in the nucleolus in a subset of cells and undergoes rapid exchange with Gag proteins in the nucleus (Lochmann et al., 2013). However, we had not reported the characteristics of Gag proteins localized to nucleoplasmic puncta. To examine the kinetic properties of Gag.L219A localized within nucleoplasmic foci, we used live-cell confocal imaging to examine the movement of the foci over a 10 min time period with z-stacks acquired every 8 s (Figure 1C). Following acquisition, the x, y, and z coordinates of each of 149 foci that were tracked during the entire 10 min imaging period were analyzed to measure particle movement. The mean square displacement (MSD) over time of each particle track was analyzed and a curve was fitted to the data (Tarantino et al., 2014). Using stringent parameters for curve fitting (*R*^2^ > 0.8) of each particle track, the data from 149 Gag nuclear foci were further analyzed for their distance of diffusion. The average anomalous diffusion coefficient (α) of these tracked particles was found to be 0.46 ± 0.16 with high significance for α values < 1.0 (*p* = 1.4 × 10^−82^), indicating a pattern of obstructed diffusion based on definitions previously described by Bacher and colleagues (confined diffusion, α < 0.1; obstructed diffusion, 0.1 ≤ α < 0.9; simple diffusion, 0.9 ≤ α < 1.1; directed motion, α ≥ 1.1) (Bacher et al., 2004). The α values were binned to plot a histogram (Figure 1D), which indicated that 100% of nuclear Gag foci display obstructed diffusion, indicating that the movement of each Gag focus has a limited range of movement, suggesting that Gag molecules may be tethered to a cellular partner within the nucleus.

### Analysis of Gag.L219A co-localization with host proteins in subnuclear bodies

**Figure 2:**
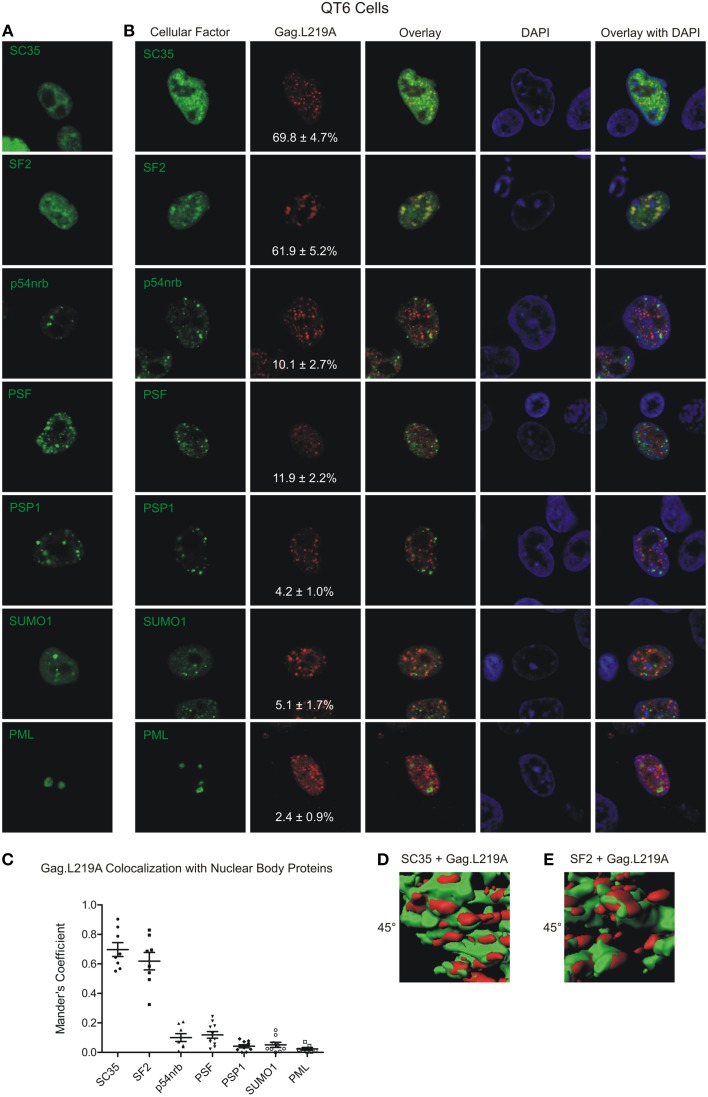
**Localization of Gag.L219A with host nuclear body proteins in QT6 cells. (A)** Localization of Gag.L219A and nuclear body proteins in singly transfected QT6 cells. **(B)** Co-localization analysis between Gag.L219A and the indicated nuclear body proteins in co-transfected QT6 cells. Merging of Gag.L219A and nuclear body marker protein channels is displayed (Overlay). The DAPI channel is also depicted. The percentage of Gag.L219A with each factor is depicted in the Gag.L219A channel with the standard error of the mean. **(C)** Scatterplot depicting the mean and standard error of the mean of Gag.L219A co-localization with each of the nuclear body protein. **(D)** Still image from Supplemental Video 1 that closely examines a surface rendering of SC35 (green) and Gag.L219A (red). **(E)** Still image from Supplemental Video 2 that closely examines a surface rendering of SF2 (green) and Gag.L219A (red).

The particle tracking data suggest that Gag.L219A foci appeared to be tethered in foci which resemble subnuclear bodies. To determine whether Gag.L219A co-localized with host proteins in subnuclear bodies, we expressed fluorescently-tagged splicing factors that are components of speckles (SC35 and SF2); paraspeckles (PSP1, PSF, and p54nrb); or PML bodies (PML and SUMO1). Plasmids expressing these nuclear body components tagged with YFP were used to co-transfect avian (QT6) and human (HeLa) cells with pGag.L219A-CFP. We expected human SC35 and SF2 to form characteristic splicing speckles in QT6 cells due to the high level of conservation between human and chicken orthologs (98.19 and 98.79% amino acid identity, respectively). However, SC35 and SF2 appeared more diffuse in QT6 cells even when a low amount of plasmid DNA was used for transfection (100 ng), although there were areas of consolidation where the protein was concentrated (Figure 2A). Of interest, both SC35 and SF2 co-localized with Gag.L219A foci to a high degree. Quantitative Mander's analysis performed in 8 cells revealed that a mean of 69.8 ± 4.7% of Gag.L219A co-localized with SC35 and 61.5 ± 5.2% of Gag.L219A with SF2 (Figure 2C). To determine whether co-localization was present in 3-dimensions, z-stacks were obtained and reconstructions were performed using Imaris imaging analysis software (Supplemental Movie S2). Rendering of the Gag.L219A (red) and SC35 (green; Figure 2D, left) or SF2 (Figure 2D, right) signals revealed that Gag.L219A/SC35 and Gag.L219A/SF2 co-localized in the x, y, and z dimensions and appear to be in close proximity, at least based on the limits of resolution of the microscopic images obtained (theoretical resolution 250 nm in the x and y planes and 600 nm in the z plane). For Gag.L219A/SC35 and Gag.L219A/SF2, the Mander's co-localization values were statistically significantly higher (*p* < 0.0001 in both cases) than the quantitative co-localization measured between Gag.L219A and proteins that reside in paraspeckles (p54nrb, PSF, and PSP1) or PML bodies (PML and SUMO1) (Figures 2B,C). Together, these data suggest that Gag.L219A protein accumulated at subnuclear locations enriched in splicing speckle components SC35 and SF2.

**Figure 3:**
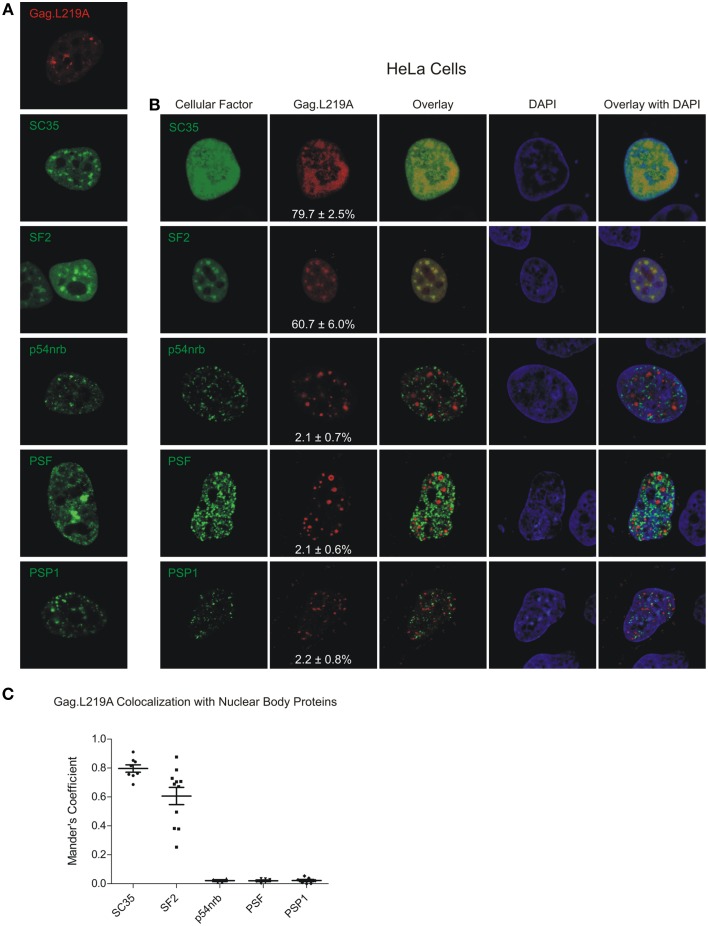
**Localization of Gag.L219A with host nuclear body proteins in HeLa cells. (A)** Localization of Gag.L219A and nuclear body proteins in singly transfected HeLa cells **(B)** Co-localization analysis between Gag.L219A and the indicated nuclear body proteins in co-transfected HeLa cells. Merging of Gag.L219A and nuclear body marker protein channels is displayed (Overlay). The DAPI channel is also depicted. The percentage of Gag.L219A with each factor is depicted in the Gag.L219A channel with the standard error of the mean. **(C)** Scatterplot depicting the mean and standard error of the mean of Gag.L219A co-localization with each of the nuclear body proteins.

Next, because we observed co-localization between Gag.L219A and human SC35 and SF2 expressed in avian cells, we wanted to determine whether similar co-localization patterns would be observed in human cells, in which the localization of splicing factors has been more extensively studied. When expressed alone, SC35 adopted its characteristic speckled appearance; however, co-expression with Gag.L219A resulted in a more diffuse pattern, and there was a high degree of co-localization between the proteins (79.7 ± 2.5% of Gag co-localized with SC35; Figures 3A,B). SF2 accumulated in speckles when expressed alone, and SF2 also showed a high degree of co-localization with Gag.L219A (60.6 ± 6%). Gag co-localization was significantly higher with SC35 compared to SF2 (*p* = 0.0192) and both SC35 and SF2 were more strongly associated with Gag.L219A compared to p54nrb, PSF or PSP1 (*p* < 0.0001). Analysis of cells co-expressing Gag.L219A and PML or SUMO1 could not be performed due to cell toxicity (data not shown).

**Figure 4:**
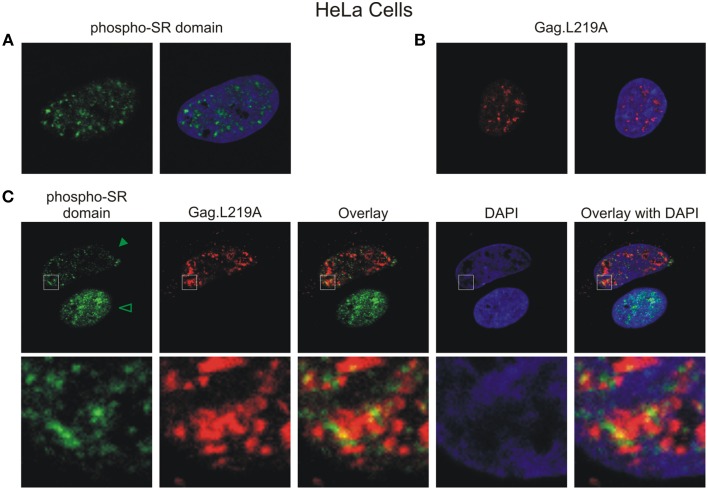
**Gag.L219A localization with endogenous splicing speckles in HeLa cells**. **(A)** Visualization of endogenous splicing speckles in HeLa cells using immunofluorescence with α-phospho RS antibody (also called α-SC35 antibody; see Materials and Methods). **(B)** Localization of Gag.L219A in unstained HeLa cells fixed and permeabilized using the conditions outlined in Materials and Methods used to visualize endogenous splicing speckles. **(C)** Gag.L219A transfected HeLa cells were stained with α-phospho RS antibody. The Gag.L219A and α-phospho RS channels were combined (Overlay). This merged image was overlaid with the DAPI channel (Overlay with DAPI). The same region from each channel denoted by the white box was cropped, enlarged, and displayed in the bottom panel. The intensity of α-phospho RS antibody decreased in the presence of Gag.L219A (indicated by solid arrowhead) compared to cells in which Gag.L219A was not expressed (indicated by open arrowhead) (*p* < 0.001; 6 cells were analyzed per condition).

To determine whether Gag.L219A foci co-localized with endogenous nuclear splicing speckles, we performed immunofluorescence using an antibody that recognizes the phosphorylated RS domains of SF2 and SC35 (Figure 4A). Gag.L219A accumulated in nuclear foci in transfected HeLa cells that appeared similar to those observed in QT6 cells (Figure 4B). In cells expressing Gag.L219A, overlapping signals were observed at the intersection of the phosphorylated SR domain proteins and Gag.L219A foci, which appeared to be juxtaposed (see enlarged image in bottom row of Figure 4C), suggesting that Gag foci form near accumulations of splicing speckle components. Of note, in HeLa cells expressing Gag.L219A, the amount of endogenous SC35/SF2 staining was dramatically reduced, as indicated by the statistically significant (*p* < 0.001) decrease in the mean signal intensity of the anti-phospho RS antibody channel (Figure 4C, solid arrowhead) compared to cells in which there was no Gag expression (open arrowhead). This result suggests that expression of Gag.L219A interferes with staining of endogenous phospho RS domains of splicing factors, although the mechanism remains unclear.

### Co-expression of Clk1 enhances co-localization of Gag.L219A with SC35 and SF2

**Figure 5:**
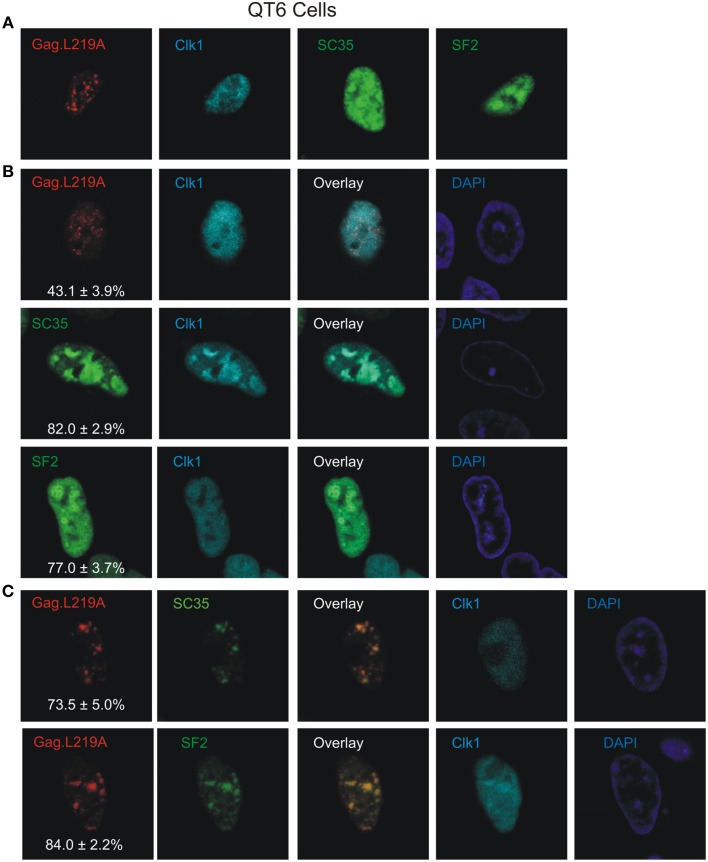
**The effect of Clk1 on Gag.L219A localization in QT6 cells. (A)** Localization of Gag.L219A, Clk1, SC35, or SF2 in singly transfected QT6 cells. **(B)** Co-expression of Clk1 with Gag.L219A, SC35, or SF2 in QT6 cells. “Overlay” depicts a merge of the Clk1 and Gag.L219A or splicing factor channels. The DAPI channel is also displayed. The percentage of Gag.L219A, SC35, or SF2 co-localizing with Clk1 is depicted in the corresponding Gag.L219A, SC35, and SF2 channels with the standard error of the mean. **(C)** QT6 cells co-transfected with Gag.L219A, Clk1, and SC35 (top panel) or SF2 (bottom panel). Merging the Gag.L219A and splicing factor channels results in the “Overlay” channel. The DAPI channel is also displayed. The percentage of Gag.L219A co-localization with SC35 or SF2 in the presence of Clk1 overexpression is depicted in the corresponding Gag.L219A channel with the standard error of the mean.

Phosphorylation is a major mechanism for regulating the localization of SR proteins in the nucleus (Yeakley et al., 1999), therefore we examined whether the degree of phosphorylation of splicing factors SC35 and SF2 influenced their association with Gag.L219A. To that end, murine Clk1, an SR protein kinase (SRPK) that phosphorylates the RS domains of SC35 and SF2 (Gui et al., 1994; Colwill et al., 1996; Nayler et al., 1997; Koizumi et al., 1999; Aubol et al., 2002; Ngo et al., 2005) was expressed as an mCherry fusion protein in QT6 cells, either alone (Figure 5A) or in conjunction with Gag.L219A-CFP, SC35-YFP or YFP-SF2 (Figure 5B). When co-expressed with Gag.L219A, there was no significant co-localization with Clk1-mCherry (Figure 5B, upper row). However, as expected, there was co-localization between Clk1/SC35 and Clk1/SF2 (Figure 5B, lower rows) because Clk1 phosphorylates both SC35 and SF2.

In cells expressing Gag.L219A/Clk1/SC35 (Figure 5C, upper row), the degree of Gag co-localization with SC35 increased to 73.5% ± 5% (compared to 69.8% without Clk1, Figure 2), although the increase was not statistically significant. However, Clk1 co-expression did significantly increase the co-localization of Gag.L219A with SF2 to 84% ± 2.2% (compared to 62.9% without Clk1, Figure 2; *p* = 0.0066). To assess whether Clk1 hyperphosphorylated YFP-SF2 and SC35-YFP in QT6 cells, we performed Western blotting of nuclear lysates (Supplemental Figure 1). For both SF2 and SC35, there was a change in the migration of the hyperphosphorylated proteins (red asterisks) in cells co-expressing Clk1-mCherry compared to the position of YFP-SF2 and SC35-YFP isolated from cells not expressing Clk1-mCherry (red circles). Treatment of the nuclear lysates with calf intestinal phosphatase dephosphorylated the proteins, as demonstrated by the faster migration of phosphatase-treated forms of SF2 and SC35, consistent with previous reports (Ngo et al., 2005). Together, these results suggest that the association of Gag.L219A with splicing factors, particularly SF2, was enhanced by hyperphosphorylation of the RS domain.

### The number of nuclear Gag.L219A foci increases with SC35 overexpression

**Figure 6:**
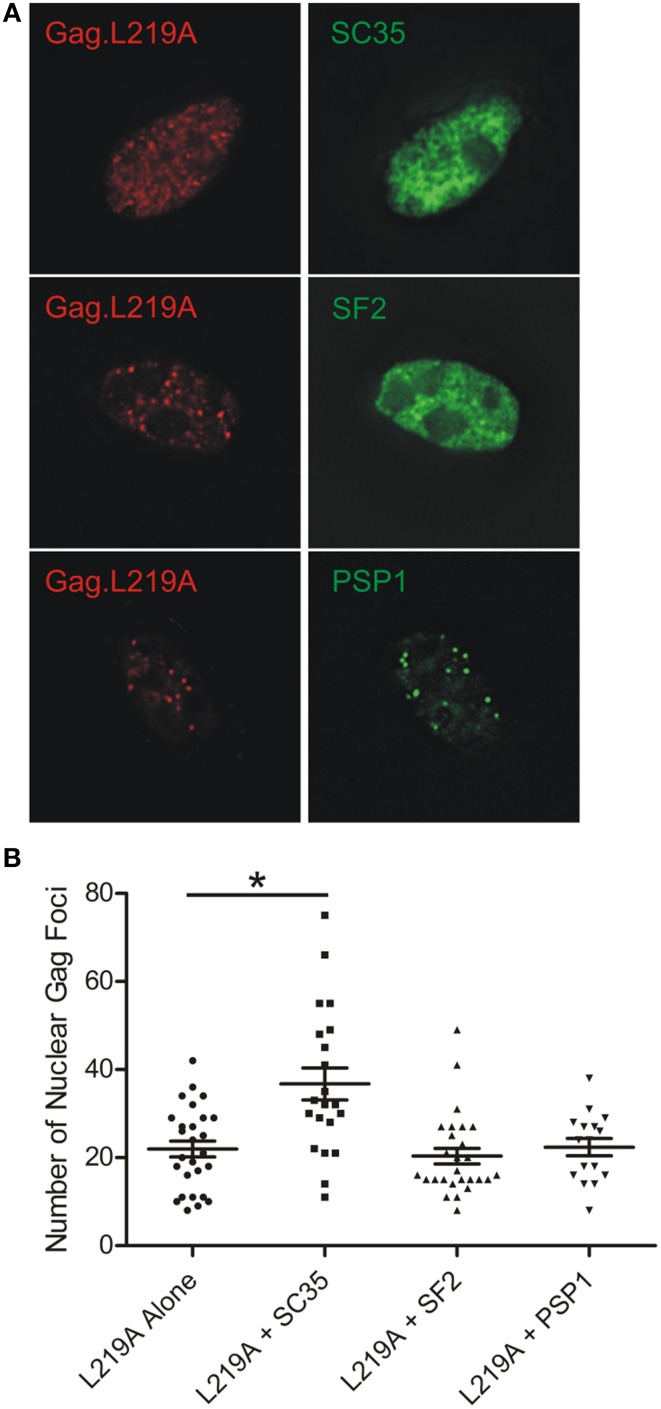
**Quantification of Gag.L219A nuclear foci number with co-expression of SC35, SF2, or PSP1. (A)** Representative images of QT6 cells co-transfected with Gag.L219A and SC35, SF2, or PSP1. **(B)** Quantification of the number of Gag.L219A nuclear foci in QT6 cells expressing Gag.L219A alone or Gag.L219A co-transfected with SC35, SF2, or PSP1. Mean and standard error of the mean are indicated for each transfection. Nuclear foci in at least 16 QT6 nuclei were counted. ^*^denotes *p* = 0.0003.

During the course of our imaging studies, we noticed that the number of nuclear Gag.L219A-CFP foci appeared to increase in cells that co-expressed SC35-YFP. To determine whether this effect was specific for SC35, we compared the number of nuclear Gag.L219A foci in cells expressing Gag.L219A-CFP alone compared to cells transfected with equal amounts of pSC35-YFP, pYFP-SF2, or pYFP-PSP1 (Figure 6A). In cells expressing Gag.L219A alone, the average number of Gag foci was approximately 22 per nucleus, whereas upon co-expression of SC35-YFP, the average number of Gag nucleoplasmic foci increased significantly to 36 (Figure 6B, *p* = 0.0003). By contrast, co-transfection of equal amounts of pGag.L219A with pYFP-SF2 or pYFP-PSP1 did not lead to a significant change in the number of nuclear Gag foci. This experiment was repeated in DF1 cells with the same outcome, indicating that the result was not specific to QT6 cells (data not shown).

## Discussion

The biological role of retroviral Gag proteins in the nucleus is not well understood, even though several Gag proteins have been observed within the nucleus (Parent, 2011; Stake et al., 2013). RSV is unique among retroviral Gag proteins because the protein accumulates in the nucleus when nuclear export is blocked by treatment of cells with LMB and a CRM1-mediated nuclear export signal (NES) was identified in the p10 region of Gag (Scheifele et al., 2002, 2005). The NES mutant Gag.L219A is a very informative tool, permitting the examination of the intranuclear activities of Gag, which are difficult to study using the wild-type Gag protein because a small amount of Gag protein is present in the nucleus. In this work, we focused on understanding the characteristics of the nuclear Gag population and to identify potential interacting partners within the nucleus.

With careful inspection, the wild-type Gag protein can be detected within small nucleoplasmic foci (Figure 1B). The Gag.L219A protein accumulated in similar nuclear puncta, although the foci were larger and more numerous. The foci formed by nuclear-restricted Gag proteins exhibited obstructed diffusion with an average anomalous diffusion coefficient of 0.46, suggesting that Gag.L219A proteins are tethered to another molecule within the nucleus. In an attempt to identify the tethering partner, when we examined whether Gag.L219A was associated with other subnuclear bodies that form intranuclear foci. We found a high degree of co-localization with splicing factors SC35 and SF2, although the co-localization was partial (~70%), suggesting that either Gag proteins move dynamically between SF2/SC35 nuclear aggregates and other nucleoplasmic sites, or a different molecule could be the Gag anchor.

Finding that increased expression of SC35 induced an increase in the number of Gag foci suggests that SC35 may facilitate the formation of Gag.L219A nuclear puncta (Figure 6). Additional experiments will need to be performed to examine whether SC35 is required for the formation of Gag.L219A foci, whether Gag interacts with SC35 and if so, whether the interaction is direct or indirect. SF2 also co-localized with Gag foci (Figure 2), and the degree of co-localization increased with co-expression of the with SR protein kinase Clk1, which phosphorylates the RS domain of splicing factors (Colwill et al., 1996). These data suggest that Gag.L219A interacts more efficiently with phosphorylated splicing factors, although alternatively, it remains possible that Clk1 has pleiotropic effects on cellular proteins that result in increased co-localization of Gag.L219A with SF2 and SC35. Furthermore, we noted that SC35 and SF2 appeared more diffuse in the nuclei of QT6 cells rather than forming discrete puncta characteristic of splicing speckles in HeLa cells (Figure 3). The localization of SR proteins is dependent on phosphorylation of their RS domains by SRPKs, including Clk1. Hyperphosphorylation of the RS domain of SF2 by Clk1 relocalizes SF2 from discrete nuclear speckles to the nucleoplasm (Ngo et al., 2005), and other work has shown that phosphorylation of the SR domain targets splicing factors to nascent RNA transcripts (Misteli et al., 1998; Yeakley et al., 1999). Therefore, the increased association of Gag.L219A with hyperphosphorylated SR proteins implies that Gag.L219A may preferentially associate with SR proteins that are primed for splicing nascent transcripts at sites of transcription.

To explain the difference in appearance of nuclear foci formed by SF2 and SC35 in avian cells compared to human cells, one possibility is that avian SRPKs do not properly phosphorylate human splicing factors (Gui et al., 1994; Colwill et al., 1996; Nayler et al., 1997; Koizumi et al., 1999), causing them to adopt a more diffuse localization. This idea is supported by the observation that expression of Clk1 in QT6 cells was associated with a more focal consolidation of Gag.L219A with SC35 and SF2 (Figure 5C). However, we cannot rule out the possibility that SC35 and SF2 require another host factor (protein or RNA) to form splicing speckles in human cells (e.g., the long noncoding RNA MALAT1) (Tripathi et al., 2010; Nakagawa et al., 2012), which may not be present in avian cells. Whereas our data suggests that Gag may co-localize with splicing factors SC35 and SF2, whether these associations are at canonical speckles near sites of transcription will require further examination.

Using a monoclonal antibody directed against the phosphorylated RS domains of splicing factors to stain for endogenous proteins, we observed that Gag.L219A and endogenous phosphorylated splicing factors appeared to be juxtaposed in HeLa cells (Figure 4C). Strikingly, immunostaining with the anti-phospho RS domain antibody was markedly reduced in HeLa cells that also co-expressed Gag.L219A (Figure 4C). These findings combined with the 3-dimensional reconstructions (shown in Figure 2 and Supplemental Movies S1, S2) showing the close proximity of Gag.L219A with SC35 and/or SF2 raises the possibility that Gag may associate with splicing factors in splicing speckles, although more experiments need to be performed to test this idea. The NC domain of Gag.L219A is required for Gag to form intranuclear foci and NC also mediates Gag-Gag and Gag-RNA interactions (Kenney et al., 2008). Therefore, we must consider the idea that the Gag nuclear tether could be a host RNA; thus, it is possible that Gag.L219A interacts with SC35 and/or SF2 through an RNA-mediated association.

Why might RSV Gag interact with splicing factors in the nucleus? Considering our previous data linking nuclear localization of Gag with efficient genomic RNA packaging (Scheifele et al., 2002; Garbitt-Hirst et al., 2009), we hypothesize that RSV Gag might enter the nucleus to package the viral unspliced RNA genome shortly after it is synthesized. This strategy would target Gag to the transcription site, which is where the highest concentration of genome-length RNA is present in the cell. Additionally, Gag would have access to viral RNA before it could be spliced and could select the unspliced RNA as genome rather than permitting its use as an mRNA. We propose that RSV Gag could enter the nucleus, localizing at the periphery of speckles near transcription factories (Sutherland and Bickmore, 2009) to gain access to nascent unspliced viral RNA to capture it for packaging into virions. Other potential reasons for Gag to localize near splicing factors include altering the splicing pattern of host or viral RNAs or modifying nuclear export of viral or host RNAs.

As a means to target the unspliced RSV RNA for packaging by Gag, we propose that the NRS (negative regulator of splicing), a cis-acting element in the *gag* coding region, may play an important role. The RSV NRS regulates the balance between spliced and unspliced RSV RNA (Arrigo and Beemon, 1988; McNally and Beemon, 1992) by binding to SFp30a/b (a complex of SC35 and SF2) and U11/U12 snRNPs to form a nonfunctional spliceosome that inhibits the upstream RSV 5′ splice site (Gontarek et al., 1993; McNally and McNally, 1996). Interestingly, the RSV psi packaging sequence is located on both the spliced and unspliced viral RNA, yet the unspliced RNA is preferentially packaged by Gag into new virions. Thus, we speculate that the downstream pseudo-spliceosome assembled on the NRS could “mark” the unspliced RNA as a potential genome. Gag could interact with SC35 and SF2 bound to the NRS, scan the RNA for the high affinity psi sequence, and ultimately select an RNA containing both an NRS and psi as the genomic RNA. Additionally, it is feasible that RSV Gag associates with splicing factors to influence the global splicing program of the cell to benefit virus replication.

Interestingly, numerous splicing factors were identified as potential binding partners of the HIV-1 Gag protein using mass spectrometry (Engeland et al., 2011, 2014), including PRPF3 and PRPF4 (components of the U4/U5/U6 tri-snRNP complex), SFRS1 (SF2), SFRS2 (SC35), SFRS3 (SRp20), SRSF5 (SRp40), SRSF6 (SRp55), SFSR7 (9G8), SFRS9 (SRp30c), SRPK1 (an SR Protein Kinase). Additional investigation is required to address whether HIV-1 Gag associates with these factors in cells and whether splicing factors play a functional role in the replication cycle beyond their influence on regulating alternative splicing of retroviral RNAs.

### Conflict of interest statement

The authors declare that the research was conducted in the absence of any commercial or financial relationships that could be construed as a potential conflict of interest.
