# Supplementary material for: Interplay between the alpharetroviral Gag protein and SR proteins SF2 and SC35 in the nucleus
Source: Front Microbiol. 2015 Sep 8;6:925. doi: 10.3389/fmicb.2015.00925 (PMC4562304; doi:10.3389/fmicb.2015.00925)
Supplement: Supplementary file 3 [file Supp.LegendsandMethods.DOCX]

**Supplemental Movie 1: Gag.L219A with SC35-YFP.** A volume rendering of a QT6 cell expressing Gag.L219A (red) and SC35 (green) was generated using Imaris software. Animation begins with 360-degree rotation around the *y-axis* displaying the Gag.L219A channel, followed by a 360-degree rotation around the same axis displaying the SC35 channel. The Gag (red) and SC35 (green) channels were then merged. Next, the Gag.L219A channel was rendered as a surface and rotated, followed by a surface rendering of SC35. Both of the surface renderings were combined and rotated 360-degrees.

**Supplemental Movie 2: Gag.L219A with YFP-SF2.** A volume rendering of a QT6 cell expressing Gag.L219A (red) and SF2 (green) was generated using Imaris software. Animation begins with 360-degree rotation around the *y-axis* displaying the Gag.L219A channel, followed by a 360-degree rotation around the same axis displaying the SF2 channel. The Gag (red) and SF2 (green) channels were then merged. Next, the Gag.L219A channel was rendered as a surface and rotated, followed by a surface rendering of SF2. Both of the surface renderings were combined and rotated 360-degrees.

**Supplemental Figure 1: Clk1 hyperphosphorylation of SF2 and SC35 in avian cells.** Western blotting analysis of nuclear lysates was performed to examine the phosphorylation status of splicing factors SC35-YFP and YFP-SF2 in the absence or presence of co-transfected pClk1-mCherry. YFP-SF2 and SC35-YFP were detected using α-GFP antibody and HRP-conjugated secondary antibody. A portion of each sample was treated with calf intestinal phosphatase (CIP) as indicated by (+) or (–) to dephosphorylate the protein. The red closed circle indicates the center of the band for SF2 or SC35 in cells without Clk1-mCherry (phosphorylated form). The bands labelled with the red asterisk are aligned with the center of the SF2 or SC35 bands in cells expressing Clk1-mCherry (hyperphosphorylated forms).

**Supplemental Figure Methods:** 0.6 x 10^6^ QT6 cells were seeded in 60mm dishes and transfected using the calcium phosphate method with the indicated DNA constructs. Cells were collected 18 hours post transfection and nuclear extracts were prepared using a modification of a procedure described previously ([Schreiber et al., 1989](#_ENREF_1)). Specifically, cells were scraped into 1.5 ml of cold PBS and pelleted at 800xg rpm for 5 minutes. The supernatant was removed and the cell pellet was resuspended in 200 μl ice-cold lysis buffer [(10 mM HEPES, pH 7.9, 10 mM KCl, 0.1mM EDTA, 0.4% Nonidet P-40, and 1X EDTA-free protease inhibitor cocktail (Roche)] by pipetting up and down 10 times, followed by a 5 minute incubation on ice. The samples were centrifuged 800 x *g* for 5 minutes, and the supernatants (cytosol) were discarded. Pelleted nuclei were resuspended in 100 μl of nuclear extraction buffer (10 mM HEPES, pH 7.9, 0.4 M NaCl, 1 mM DTT (Dithiothreitol), 100 U/ml OmniCleave nuclease (Epicentre), 1X EDTA-free protease inhibitor cocktail) and incubated for 10 minutes at 37°C. Following nuclease incubation, 2 mM EGTA was added and tubes were vortexed for 15 seconds. The samples were rotated for 15 minutes at 4°C followed by a high-speed spin for 10 minutes in a microcentrifuge. A portion (50 μg) of each sample was treated with 1 U/μg of calf intestinal phosphatase (New England Biolabs) for 1 hour at 37°C. Samples were mixed with 4X SDS loading buffer (250 mM Tris pH 6.8, 40% glycerol, 8% β-mercaptoethanol, 0.4% w/v bromophenol blue, 8% SDS, and 50 mM DTT) and boiled for 10 minutes prior to loading on a 10% SDS-PAGE gel. Proteins were detected using α-GFP antibody (Abcam ab290) and HRP-conjugated secondary antibody.

**Supplemental reference:**

Schreiber, E., Matthias, P., Müller, M.M., and Schaffner, W. (1989). Rapid detection of octamer binding proteins with 'mini-extracts', prepared from a small number of cells. *Nucleic Acids Research* 17**,** 6419.
