# Supplementary figures and images for: Interplay between the alpharetroviral Gag protein and SR proteins SF2 and SC35 in the nucleus
Source: Front Microbiol. 2015 Sep 8;6:925. doi: 10.3389/fmicb.2015.00925 (PMC4562304; doi:10.3389/fmicb.2015.00925)

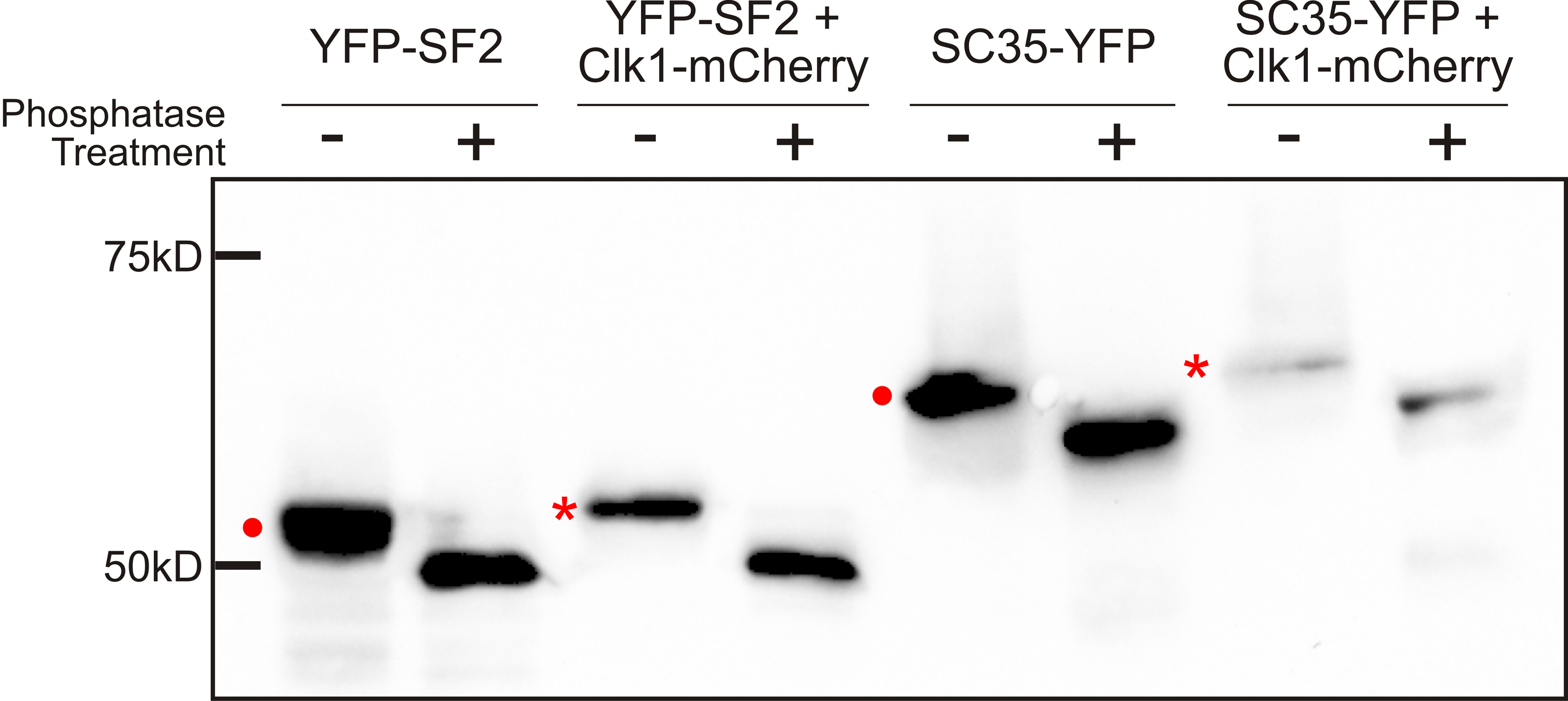

Supplement: Supplementary file 4 [file Figure1.TIF]
